# Nutritional composition of human milk and its association with maternal and perinatal factors

**DOI:** 10.1590/1984-0462/2024/42/2023001

**Published:** 2023-11-24

**Authors:** Daniele Marano, Raquel Ximenes Melo, Danielle Aparecida da Silva, Marina Machado Vilarim, Maria Elisabeth Lopes Moreira

**Affiliations:** a Fundação Oswaldo Cruz, Instituto Nacional de Saúde da Mulher, da Criança e do Adolescente Fernandes Figueira – Rio de Janeiro, RJ, Brazil.

**Keywords:** Human milk, Postpartum, Perinatal, Maternal, Maternity, Newborn, Leite humano, Pós-parto, Perinatal, Materno, Maternidade, Recém-nascido

## Abstract

**Objective:**

The objective of this study was to evaluate the influence of maternal and perinatal factors on the nutritional composition of human milk.

**Methods:**

A cross-sectional study was conducted between November 2018 and January 2020, with 181 donors selected in Tertiary Health Units of the Unified Health System — from one collection station and five Human Milk Banks. Data were collected through a standardized questionnaire. To be fit to be a donor in a Human Milk Banks and produce mature milk were the eligibility criteria to participate in the study. We excluded milk samples with Dornic acidity above 8° D. The dependent variables were the macronutrients of human milk (i.e., carbohydrates, proteins, lipids, and total energy), that were analyzed using spectroscopy with the Miris Human Milk Analyzer™. The maternal and perinatal factors were the independent variables.

**Results:**

Women with pre-gestational obesity and gestational weight gain above the recommendation showed a lower protein concentration compared to eutrophic women (median=0.8, interquartile range (IQR): 0.7–0.9 vs. median=0.8, and IQR: 0.8–1.0) and those with adequate gestational weight gain (median=0.8, IQR: 0.7–0.9 vs. median=0.9, and IQR: 0.8–1.0), respectively. The other analyzed factors (i.e., maternal habits, comorbidities, and perinatal factors) were not associated with the nutritional composition of human milk.

**Conclusions:**

The assessment of factors associated with the nutritional composition of human milk is extremely important to assist postpartum care. Pre-gestational obesity and inadequate gestational weight gain were the only factors statistically associated with the nutritional composition of human milk as they impacted its protein content.

## RESUMO

**Objetivo:**

Avaliar a influência de fatores maternos e perinatais na composição nutricional do leite humano.

**Métodos:**

Estudo transversal, realizado entre novembro de 2018 e janeiro de 2020, com 181 doadoras selecionadas em Unidades Terciárias de Saúde do Sistema Único de Saúde de um Posto de Coleta e cinco Bancos de Leite Humano. Foram coletados dados das variáveis maternas e dos recém-nascidos por meio de questionário padronizado. Estar apta para ser doadora em um Banco de Leite Humano e produzir leite maduro foram os critérios de elegibilidade para participar do estudo. As amostras de leite com acidez Dornic acima de 8° D foram excluídas. Os macronutrientes do leite humano, variáveis dependentes (carboidratos, proteínas, lipídeos, energia total) foram analisados pela técnica de espectroscopia de transmissão no infravermelho médio com o Miris Human Milk Analyzer™. Os fatores maternos e perinatais foram as variáveis independentes.

**Resultados:**

Mulheres com obesidade pré-gestacional e ganho de peso gestacional acima do recomendado apresentaram menor concentração proteica em relação às eutróficas (mediana=0,8, intervalo interquartil [IQR]: 0,7–0,9 *vs*. mediana=0,8, IQR: 0,8–1,0) e aquelas com ganho de peso gestacional adequado (mediana=0,8, IQR: 0,7–0,9 *vs*. mediana=0,9, IQR: 0,8–1,0), respectivamente. Os demais fatores analisados (hábitos maternos, comorbidades, fatores perinatais) não se associaram com a composição nutricional do leite humano.

**Conclusões:**

A avaliação dos fatores associados à composição nutricional do leite humano é de extrema importância para auxiliar os cuidados pós-parto. A obesidade pré-gestacional e o ganho de peso gestacional inadequado foram os únicos fatores estatisticamente associados com a composição nutricional do leite humano por terem impactado o seu teor de proteína.

## INTRODUCTION

Human milk has a complex composition. In addition to lipids, proteins, and carbohydrates, it also offers immune cells and bioactive molecules. It is considered a protective agent for non-communicable diseases in adult life such as diabetes, obesity, cardiovascular diseases, and allergic diseases.^1^ The recommendation on breastfeeding is that it is exclusive in the first 6 months of life, and after that period, supplemented with other healthy foods, with breastfeeding being maintained for 2 years or more.^2^

Breastfeeding undoubtedly benefits the health of women and children and is the best strategy to reduce infant mortality.^3^ The World Health Organization (WHO), the United Nations Children’s Fund,^2^ the American Academy of Pediatrics,^4^ and the Brazilian Ministry of Health^5^ have recommended that donor pasteurized human milk is the best food substitute for newborns whenever the mother’s own milk is not available. There is still a debate on the quantities and suitability of macronutrients concerning very low birth weight newborns and premature infants.^6^

Mothers who had full-term pregnancies are chiefly the donors of human milk, milked and pasteurized in Human Milk Banks (HMB), and present important differences in composition.^7^ Such variations can be influenced by some maternal conditions, namely, the nutritional status during pregnancy, maternal age,^8^ lifestyle, quality of the diet,^9^ maternal diseases (arterial hypertension and diabetes mellitus), the lactation period,^10,11^ and the sex of the child,^12^ among others. Therefore, this study aimed to evaluate the association between maternal/perinatal factors and the nutritional composition of human milk from donors of HMB. These data will further assist the adequate milk distribution to hospitalized newborns according to their nutritional needs.

## METHOD

This is a cross-sectional study of donors from one Collection Station and four HMB in Rio de Janeiro and one HMB from Duque de Caxias. The data collection started in November 2018 and ended in January 2020, based on a non-probabilistic sample of 181 donors.

It was realized in the HMB of the Fernandes Figueira National Institute for Women, Children and Adolescent Health/Oswaldo Cruz Foundation (IFF/FIOCRUZ), which is a National Referral Center for the Brazilian Network of HMB and a Global Referral Center.

To be fit to be a donor in an HMB and produce mature milk were the eligibility criteria to participate in the study. We excluded milk samples with Dornic acidity above 8° D. A trained HMB phone operator contacted the potential donors to enquire and confirm their participation. The operator also explained the general objective of the study and in case of acceptance the subsequent procedure to collect milk at home. On the day of the home milk collection, the donor would receive at home two copies of the informed consent form signed by the principal investigator and requiring the donor’s signature. They would also receive a labeled kit for collecting samples, written guidance for the collection of a minimum volume of 40 mL, and immediate freezing of milk samples. The sample bottles were identified with registration number, name, date, milking time, and if the milk collection was before or after a feeding.

The principal investigator of the study then contacted the donor by phone and reviewed the instruction to fill out the label and read out the informed consent form and the standard milking instructions.^13^ The donor also answered a standardized questionnaire that included sociodemographic, prenatal, and maternal habits information.

The collection team returned to the donor’s house to pick up the milk samples, and these were transported in isothermal containers with recyclable ice at -1°C temperature and were stored in a freezer at the temperature of -20°C up to the time of analysis. We used the Dornic method to determine the acidity of human milk. We analyzed the samples in standard triplicates of 1 mL each and added one drop of the hydroalcoholic phenolphthalein indicator solution at 1% w/v in 95° GL alcohol. We added drops of the standard solution sodium hydroxide N/9-Dornic solution until the color changed to light pink. Of the collected 314 milk samples, 181 met the acidity criterion for subsequent analysis.^14^

We analyzed as dependent variablesof the study the macronutrients of human milk, (i.e., carbohydrates, proteins, lipids, and total energy), using the medium infrared transmission spectroscopy technique with the Miris Human Milk Analyzer™ (Miris AB, Uppsala, Sweden). The human milk sample was placed in the thermostatic bath at 40°C for 5-10 min and homogenized with the Miris Ultrasonic Processor (1.5 s/mL) (Miris Sonicator™). Before using the equipment and after every 10 samples, we repeated the calibration and adjustment procedures.

The independent variables were maternal age, alcohol intake during and after pregnancy, smoking during and after pregnancy, presence of maternal morbidities (i.e., hypertension and diabetes mellitus, both gestational and chronic), pre-gestational nutritional status, gestational weight gain, the sex of the newborn, type of delivery, and gestational age at delivery. To classify the pre-gestational nutritional status, as recommended by the Institute of Medicine,^15^ categories of the body mass index (BMI) were defined based on the cutoff points recommended by the WHO:^16^ low weight (≤18.5 kg/m^2^), eutrophic (>18.5 to ≤24.9 kg/m^2^), overweight (>25 to ≤29.9 kg/m^2^), and obese (≥30 kg/m^2^). We calculated the total gestational weight gain by subtracting pre-gestational weight (baseline) from the weight of the last prenatal consultation. In this study, we categorized the adequacy of weight gain in three groups of pre-gestational nutritional status: adequate, insufficient, and excessive as recommended by the Institute of Medicine.

The numerical variables are presented in mean values and standard deviation or as medians and percentiles 25 and 75 and categorical variables in absolute frequencies and percentages. The comparison of milk macronutrients between the independent variables in categorical form was performed using the Kruskal-Wallis with post hoc Dunnett’s multiple-comparison and Mann-Whitney tests. We used the Statistical Package of Social Sciences version 22 program for statistical analyses, and the level of statistical significance was set at 5% (p<0.05) for all analyses. The study was approved by the Research Ethics Committee of the Fernandes Figueira National Institute for Women, Children and Adolescent Health (CEP/IFF) (CAE: 97982918.5.0000.5269) and is in accordance with Resolution 466/12 of the National Health Council (CNS, 2011 – Resolution Nº 466/12). All human milk donors participating in the study signed an informed consent form (Terms of Free Informed Consent – TCLE).^17^

## RESULTS

A total of 181 samples of human milk from the donor’s milk of the Brazilian HMB Network were analyzed. The nutritional composition of mature milk of the study participants showed concentrations of lipids 2.5 g/100 mL, carbohydrates 7.7 g/100 mL, proteins 0.8 g/100 mL, and calories 57.0 kcal/100 mL (median values, Table 1).

**Table 1.** Nutritional composition of mature human milk from bank donors, 2018–2020.

	Average	Median	Percentiles 25–75	SD
Lipids (g/100 mL)	2.7	2.5	1.6–3.5	1.5
Carbohydrates (g/100 mL)	7.4	7.7	7.2–7.9	1.0
Proteins (g/100 mL)	0.9	0.8	0.8–1.0	0.3
Calories (kcal/100 mL)	59.2	57.0	49.5–67.0	14.3

The mean maternal age was 33.11 years, ranging from 16 to 43 years; 79.6% completed higher education and 64.1% declared themselves white. The mean gestational age was 39 weeks, and 56.4% had cesarean section (Table 2). Regarding maternal habits, 14.4% consumed alcohol during pregnancy and 11.6% during breastfeeding (Table 2). Regarding the pre-gestational nutritional status, 34.8% were overweight and 37% gained weight above the limits recommended by the IOM during pregnancy (Table 2).

**Table 2.** Sociodemographic, behavioral, and obstetric data of human milk donors, 2018–2020.

		Average (SD)	n	%
Maternal age (years)		33.1±5.1	–	–
Schooling	High school incomplete	–	3	1.7
	High school complete	–	26	14.4
	University incomplete	–	8	4.4
	University complete	–	144	79.6
Skin color	White	–	116	64.1
	Interracial	–	47	26
	Black	–	13	7.2
	Yellow	–	5	2.8
Marital status	Single	–	14	7.7
	Married	–	123	68
	Stable union	–	43	23.8
	Divorced	–	1	0.6
Prenatal care service	Public	–	27	14.9
	Private	–	154	85.1
Alcohol during pregnancy	Yes	–	26	14.4
Smoking during pregnancy	No	–	177	97.8
Drug consumption during pregnancy	No	–	180	99.4
Alcohol whilst breastfeeding	No	–	160	88.4
Smoking whilst breastfeeding	No	–	180	99.4
Pre-gestational hypertension	No	–	178	98.3
Hypertension during pregnancy	No	–	168	92.8
Diabetes mellitus pre-gestational	No	–	181	100
Diabetes mellitus during pregnancy	No	–	175	96.7
Pre-gestational nutritional status (WHO^16^)	Low weight	–	10	5.5
	Adequate	–	108	59.7
	Overweight	–	46	25.4
	Obese	–	17	9.4
Gestational weight gain (IOM^15^)	Low	–	55	30.4
	Adequate	–	59	32.6
	Above	–	67	37
Sex	Female	–	98	54.1
	Male	–	83	45.9
Type of delivery	Normal	–	76	41.9
	Cesarean	–	102	56.4
	Forceps	–	3	1.7
Birth weight (g)	–	3254.8±501.0	–	–
Gestational age (weeks)	–	39.1±1.4	–	–

**Figure 1.**
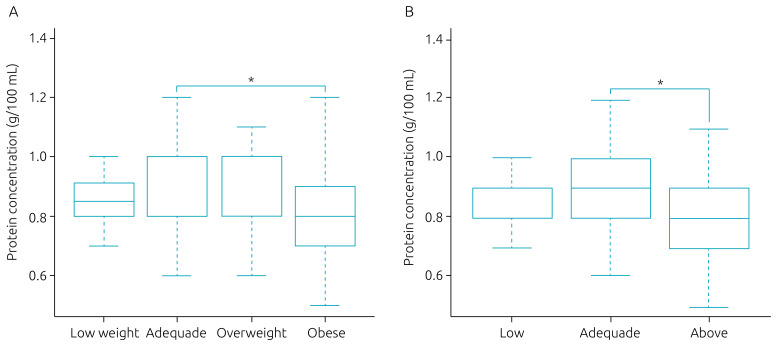
Protein concentration in human milk according to pre-gestational nutritional status and weight gain during pregnancy. (a) Pre-gestational. (b) Weight gain during pregnancy.

The nutritional status affected the concentration of proteins in human milk. Women with pre-gestational obesity and gestational weight gain above the recommendation showed a lower protein concentration compared to eutrophic women (median=0.8, interquartile range (IQR): 0.7–0.9 vs. median=0.8, and IQR: 0.8–1.0) and those with adequate gestational weight gain (median=0.8, IQR: 0.7–0.9 vs. median=0.9, and IQR: 0.8–1.0), respectively (Figure 1). The other analyzed factors (i.e., maternal habits, comorbidities, and perinatal factors) were not associated with the nutritional composition of human milk (Table 3).

*p-value<0.05 compared to “adequate” pre-gestational status (Eutrophic x Obese: p=0.017); ^†^p-value<0.05 compared to “adequate” weight gain gestational (Adequate x Above: p=0.017)

**Table 3.** Analysis of potential maternal factors associated with the nutritional composition of human milk, 2018–2020.

		n	Lipids	p-value	Carbohydrates	p-value	Proteins	p-value	Calories	p-value
			Median (percentiles 25–75)		Median (percentiles 25–75)		Median (percentiles 25–75)		Median (percentiles 25–75)	
Alcohol during pregnancy	No	155	2.4 (1.6–3.4)	0.862	7.7 (7.1–7.9)	0.842	0.8 (0.8–1.0)	0.730	57.0 (50.0–66.0)	0.762
	Yes	26	2.7 (1.3–3.6)		7.8 (7.2–7.8)		0.8 (0.8–1.0)		58.5 (48.0–69.0)	
Alcohol during breastfeeding	No	160	2.5 (1.6–3.5)	0.969	7.7 (7.1–7.9)	0.533	0.8 (0.8–1.0)	0.379	57.0 (50.0–66.5)	0.950
	Yes	21	2.6 (1.3–3.4)		7.8 (7.3–7.9)		0.8 (0.8–0.9)		59.0 (49.0–67.0)	
Smoking during pregnancy	No	177	2.5 (1.6–3.4)	0.284	7.7 (7.2–7.9)	0.739	0.8 (0.8–1.0)	0.563	57.0 (50.0–67.0)	0.453
	Yes	4	3.4 (2.4–5.4)		7.2 (6.4–8.8)		0.9 (0.8–2.6)		61.0 (54.5–96.0)	
Smoking during breastfeeding	No	180	2.5 (1.6–3.4)	–	7.7 (7.2–7.9)	–	0.8 (0.8–1.0)	–	57.0 (49.5–66.5)	–
	Yes	1	7.4		9.7		4.1		130.0	
Pre-gestational hypertension	No	178	2.5 (1.6–3.5)	0.998	7.7 (7.2–7.9)	0.475	0.8 (0.8–1.0)	0.722	57.0 (49.0–67.0)	0.907
	Yes	3	2.5 (2.2–2.8)		7.9 (7.0–8.1)		0.8 (0.7–1.0)		59.0 (52.0–64.0)	
Hypertension during pregnancy	No	168	2.5 (1.6–3.5)	0.663	7.7 (7.2–7.9)	0.648	0.8 (0.8–1.0)	0.981	57.0 (50.0–67.0)	0.703
	Yes	13	2.5 (1.7–3.0)		7.6 (7.2–8.0)		0.8 (0.8–1.0)		59.0 (49.0–65.0)	
Pre-gestational diabetes mellitus	No	181	2.5 (1.6–3.4)	–	7.7 (7.2–7.9)	–	0.8 (0.8–1.0)	–	57.0 (50.0–67.0)	–
	Yes	0	–		–		–		–	
Diabetes mellitus during pregnancy	No	175	2.5 (1.6–3.5)	0.622	7.7 (7.1–7.9)	0.805	0.8 (0.8–1.0)	0.779	57.0 (49.0–67.0)	0.692
	Yes	6	3.1 (1.7–3.3)		7.7 (7.2–7.9)		0.9 (0.8–0.9)		62.0 (51.0–66.0)	
Gestational age (weeks)	≥37	172	2.5 (1.6–3.5)	0.371	7.7 (7.2–7.9)	0.593	0.8 (0.8–1.0)	0.398	57.0 (49.5–67.0)	0.396
	34–36.9	8	2.9 (1.9–3.7)		7.6 (7.2–7.7)		1.0 (0.7–1.0)		60.5 (53.5–65.5)	
	<34	1	1.1		7.4		0.7		44.0	
Sex of the newborn	Female	98	2.5 (1.6–3.4)	0.606	7.7 (7.1–7.9)	0.858	0.8 (0.8–1.0)	0.996	56.5 (49.0–67.0)	0.521
	Male	83	2.5 (1.6–3.7)		7.7 (7.2–7.9)		0.8 (0.8–0.9)		58.0 (51.0–68.0)	

## DISCUSSION

The short- and long-term health benefits of breastfeeding to infants and mothers are indisputable.^18^ However, the association between maternal and perinatal factors and the nutritional composition of human milk, especially in HMB donors, are not conclusive. Among the maternal and perinatal factors evaluated in this study, pre-gestational obesity and gestational weight gain above the recommended limits are associated with a lower concentration of protein in mature human milk. Overweight stands out on the world stage as a serious public health problem.

By 2025, 2.3 billion adults will be overweight and more than 700 million obese in the world.^19^ Based on the data from the Brazilian Institute of Geography and Statistics,^20^ 12% of women of reproductive age are either overweight or obese. Obesity among women of reproductive age rises from 3% for women aged 18–24 years to 27.6% and 63.6% among women aged 35–44 years. This trend may have an impact on the quality and concentration of macronutrients in donated human milk.

In this study, women who were obese at the beginning of the pregnancy (9.4%) had lower protein content in the mature milk. In a longitudinal study,^21^ 55 overweight out of 66 lactating women produced mature milk with lower protein concentration when compared to eutrophic and obese women. This result does not agree with the findings of this cross-sectional study, as the decrease in protein content was observed in the milk of women with pre-gestational BMI different. Another cross-sectional study,^22^ which included 80 lactating women classified into four subgroups by age (20–30 years) and BMI (normal and overweight), observed that the protein content in milk was higher among overweight mothers aged 20 years when compared to the three other groups. The authors stated that their results might be partially explained by the interaction between age and BMI.

These variations between findings may be due to differences in the methods used for milk analyses,^23,24^ the type of milk analyzed, and the time of milk collection (before and after nursing the baby).^25^ A systematic review and meta-analysis by Leghi et al.^26^ considered inconclusive the association between maternal excess weight and the nutritional composition of human milk. In this review, some studies observed an association between the above-mentioned variables, while others found no association. As for the meta-analysis of five articles, there was no association between the protein content of mature milk and mothers’ weight, eutrophic, overweight, and obese mothers. The authors concluded that the quality of the studies made it difficult to advance the understanding of human milk composition and maternal characteristics.

In this study, milk donors with weight gain above the recommended limit had significantly lower protein content in human milk compared to women whose weight gain was adequate. Conversely, a longitudinal study of 92 women showed no association between gestational weight gain and changes in the nutritional composition of human milk.^27^ Gestational weight gain is associated with higher risks for complications during pregnancy (i.e., preeclampsia, gestational hypertension, and gestational diabetes).^28^ Weight gain during pregnancy is also associated with cephalopelvic disproportion,^29^ delay in lactogenesis II, and difficulty breastfeeding in obese women.^30^ It also affects neonatal outcomes such as shoulder dystocia in vaginal deliveries, neonatal hypoglycemia, and macrosomia, spontaneous and recommended preterm births, and cesarean delivery in nulliparous and multiparous.^31^

The association of gestational age with human milk nutritional composition has been widely studied. The results showed that the colostrum of mothers of premature infants has a higher protein content than women who had full-term births.^31^ Gidrewicz and Fenton^32^ found that the difference between the nutritional composition of the milk of mothers of premature and full-term births reduces in the course of lactation. We also found that the protein content in human milk reduces with increasing gestational age, a statistically non-significant association. There were no significant differences between gestational ages in the sample. Even though some studies have evaluated numerous factors that may influence the nutritional composition of human milk, the results are still inconclusive and limited, except for gestational age. In addition, this study seems to be a precursor to further investigations of donors of HMB.

In this research, to elucidate which factors influence the nutritional composition of donated human milk, mid-infrared transmission spectroscopy was used, which separately presents the values of each macronutrient; however, this equipment has a high cost.^33^ HMB uses crematocrit as a method of analyzing LH fat content and energy density as it is cheap, easy to perform, and reliable.^34^

Proteins are directly or indirectly linked to gastric emptying and, consequently, infant food frequency and adiposity, showing their macronutrient importance in infant nutrition.^35^

The limitations of the study are non-random sample and sample imbalance regarding the small sample size of women with certain characteristics of interest, such as hypertension, diabetes, or smokers, and the larger number of white women with a university degree, which made some comparisons unfeasible from a statistical point of view. Despite this, the results of this study may help to expand the understanding of the variability in the composition of donated HM, adjusting the needs of hospitalized babies with the pasteurized milk available in HMB.

This study represents an effort to put forward research gaps around new factors associated with nutritional composition such as pre-gestational nutritional status and gestational weight gain. We draw attention to the importance of assessing nutritional status and gestational weight gain as a high priority in perinatal and prenatal care. This is a feasible means to promote the long-term health benefits of breastfeeding to women’s health and their offspring. Moreover, the elucidation of these factors is an important guiding tool for the clinical management of HMB professionals.
